# Cyberpsychopathy: A Multidimensional Framework for Understanding Psychopathic Traits in Digital Environments

**DOI:** 10.3390/ejihpe15060107

**Published:** 2025-06-10

**Authors:** Alexandre Hudon, Emmy Harvey, Sandrine Nicolas, Mathieu Dufour, Caroline Guérin-Thériault, Julie Bérubé-Fortin, Isabelle Combey, Yu Chen Yue, Antoine Perreault, Stéphanie Borduas Pagé, Véronique MacDermott

**Affiliations:** 1 Department of Psychiatry, Institut Universitaire en Santé Mentale de Montréal, Montreal, QC H1N 3M5, Canada; stephanie.borduas-page.cemtl@ssss.gouv.qc.ca; 2 Centre de Recherche de l’Institut Universitaire en Santé Mentale de Montréal, Montreal, QC H1N 3V2, Canada; 3 Institut National de Psychiatrie Légale Philippe-Pinel, Montreal, QC H1C 1H1, Canada; mathieu.dufour.med@ssss.gouv.qc.ca (M.D.); caroline.guerin-theriault.med@ssss.gouv.qc.ca (C.G.-T.); julie.berube-fortin.med@ssss.gouv.qc.ca (J.B.-F.); isabelle.combey.med@ssss.gouv.qc.ca (I.C.); yuchen.yue.med@ssss.gouv.qc.ca (Y.C.Y.); antoine.perreault.med@ssss.gouv.qc.ca (A.P.); veronique.macdermott@umontreal.ca (V.M.); 4 Department of Psychiatry and Addictology, Faculty of Medicine, Université de Montréal, Montreal, QC H3T 1J4, Canada; emmy.harvey@umontreal.ca (E.H.); sandrine.nicolas@umontreal.ca (S.N.); 5 Department of Psychiatry, Douglas Mental Health University Institute, Montreal, QC H4H 1R3, Canada

**Keywords:** psychiatry, aversive personality traits, online aggression, trolling, digital behavior, social media, psychopathy, cyberbullying, emotion dysregulation, digital mental health

## Abstract

The rapid expansion of digital communication platforms has created new spaces for antisocial, manipulative, and emotionally detached behaviors. While psychopathy has been extensively studied in clinical and forensic settings, its digital manifestation, referred to as cyberpsychopathy, remains conceptually underdefined. This integrative review aimed to synthesize empirical research exploring psychopathy and aversive personality traits in online contexts to identify key conceptual domains and propose a preliminary definition. A systematic search across five databases yielded 35 peer-reviewed studies meeting the inclusion criteria. Using a biopsychosocial framework and thematic synthesis, six interrelated domains were identified: online behaviors (e.g., trolling and deception), online environments (e.g., anonymity and reward mechanisms), sociodemographic factors (e.g., age and gender), personality traits (e.g., psychopathy and narcissism), psychological factors (e.g., emotion dysregulation and low self-esteem), and motivations (e.g., dominance and emotional compensation). These domains interact to shape how psychopathic tendencies manifest online. Most studies were of moderate-to-high methodological quality, though variability limited direct comparisons. We propose cyberpsychopathy as a multidimensional construct representing the expression of aversive traits facilitated by digital affordances and psychological vulnerabilities. This review provides a foundational framework for understanding cyberpsychopathy and underscores the need for empirical validation and the development of assessment tools suited to digital behavior in both clinical and forensic settings.

## 1. Introduction

As digital platforms have become essential to contemporary social, educational, and professional life, there has been an increasing awareness of the frequency of mental health problems associated with online conduct (Beyari, 2023; Khalaf et al., 2023; Karim et al., 2020). The internet’s widespread use has revolutionized communication and interaction, providing unmatched convenience but also posing new mental health hazards (Naslund et al., 2020; Pantic, 2014). According to research, excessive or maladaptive online behavior is correlated to and may contribute to the development or worsening of preexisting mental health problems like anxiety and depression over time (Keles et al., 2020). Psychological distress, poor emotional control, and strained interpersonal connections have all been repeatedly associated with behaviors like obsessive internet use, cyberbullying, and problematic social media involvement (Kowalski et al., 2014; Gioia et al., 2021). These difficulties are exacerbated by the anonymity and depersonalization provided by digital contexts, which permit unrestrained and frequently damaging behaviors like trolling, harassment, and deception that might not normally take place in face-to-face interactions (Suler, 2004).

Interestingly, some maladaptive personality traits, such as impulsiveness, anger, and a lack of empathy, all of which are characteristics of psychopathy and part of the Hare Framework, seem to be amplified in the online environment (Hare et al., 2000). When exhibited on digital platforms, these characteristics show up as enduring antisocial tendencies such as taking advantage of other people, acting maliciously, and exhibiting a flagrant disrespect for social norms, all of which add to what scholars have heterogeneously referred to as violence in cyberspace or “cyberpsychopathy” (Nevin, 2015). This new concept aims to depict the distinct ways that psychopathic tendencies manifest and intensify in virtual environments. For example, cyberpsychopathy may include actions like planning phishing scams, disseminating false information, or using social engineering tactics to take advantage of people for one’s own benefit, all while avoiding accountability and detection by remaining anonymous online (Peebles, 2014). These parameters, grouped together, highlight the significant effects of online conduct on mental health and the general well-being of society. Furthermore, using a biopsychosocial approach, cyberpsychopathy likely arises from interactions between biological predispositions (e.g., impulsivity), psychological vulnerabilities (e.g., empathy deficits), and digital affordances (e.g., anonymity).

It is important to understand the complex interactions between personality traits and online settings, especially in light of our growing reliance on digital platforms for everyday tasks (van de Weijer & Leukfeldt, 2017; Basharpoor et al., 2024). In addition to providing insights into the psychological bases of harmful online behaviors, research into cyberpsychopathy lays the groundwork for the creation of focused interventions that lessen their consequences. Research is also needed to promote healthier, more moral online communities and to address the psychological hazards connected to digital interactions (Latha et al., 2020; Torous et al., 2021; K. A. Smith et al., 2023).

Furthermore, in the digital age, cyberviolence, which includes a variety of damaging practices like doxing, cyberstalking, cyberbullying, and online harassment, has become a major social issue (Aboujaoude & Savage, 2023; Chen et al., 2019). Forensic psychiatry is essential in tackling the various components of cyberviolence and comorbid psychiatric disorders, especially when it comes to understanding the psychological characteristics of offenders and evaluating the effects on victims (McGrath & Casey, 2002). The Dark Tetrad of personality (Machiavellianism, sadism, psychopathy, and narcissism) is frequently displayed by perpetrators of cyberviolence, which raises concerns about their motivations and recidivism risk factors (Pineda et al., 2022; Schade et al., 2021). Forensic psychiatrists also play a significant role in assessing the mental health effects of prolonged online attacks on victims, who often suffer from anxiety, depression, trauma, and social disengagement (Elizondo et al., 2019). Additionally, forensic psychiatrists support legal proceedings by offering expert testimony on the psychological foundations of cyberviolence and by assessing intent and responsibility (Vale et al., 2024). The integration of forensic psychiatry into legal, preventive, and rehabilitative frameworks is needed to help reduce the impact of cyberviolence on people and society as it continues to change in line with technological improvements. A clearer conceptualization of cyberpsychopathy could help forensic psychiatrists more accurately assess the psychological motivations, degrees of planning or impulsivity, and emotional detachment involved in digital offenses, thereby informing judgments about criminal responsibility, recidivism risk, and amenability to rehabilitation. Importantly, this construct is not intended as a novel personality trait, but rather as a context-dependent manifestation of established interpersonal, affective, and behavioral traits, particularly psychopathy, when filtered through the affordances of the digital environment (including anonymity, asynchronicity, deindividuation, and algorithmic reinforcement). Much like how substance use or trauma exposure can trigger latent vulnerabilities in at-risk individuals, digital spaces may catalyze the expression of latent psychopathic tendencies by removing social friction, reducing accountability, and amplifying reward-driven behaviors.

Importantly, cyberpsychopathy, while not intended as a new diagnostic category, serves as a specific operational framework designed to capture patterns of digitally mediated behaviors rooted in psychopathic traits. Unlike general constructs such as ‘online disinhibition,’ which broadly address reductions in social constraints online, or the ‘Dark Tetrad online,’ which describes the mere presence of certain aversive personality traits in digital contexts, cyberpsychopathy specifically integrates biological (trait-based vulnerabilities such as impulsivity and empathy deficits), psychological (emotional regulation difficulties and motivational factors), and social factors (platform-specific affordances like anonymity and algorithmic reinforcement) into a coherent, measurable construct. This integration allows for clear operational criteria and the precise delineation of boundaries, facilitating targeted assessment and intervention strategies.

The main objective of this integrative review is to identify key concepts of a construct to be named as cyberpsychopathy. Using these concepts, the secondary goal is to offer a preliminary definition of cyberpsychopathy. We hypothesize that cyberpsychopathy is defined by the emergence of psychopathic characteristics in online settings, including impulsiveness, lack of empathy, manipulativeness, and antisocial behavior. This phenomenon is made possible by digital anonymity and the disinhibition effect of virtual interactions. This study intends to outline the precise psychological, behavioral, and environmental components that characterize cyberpsychopathy by identifying essential themes through the review of the existing literature.

## 2. Materials and Methods

### 2.1. Search Strategies

**Figure 1:**
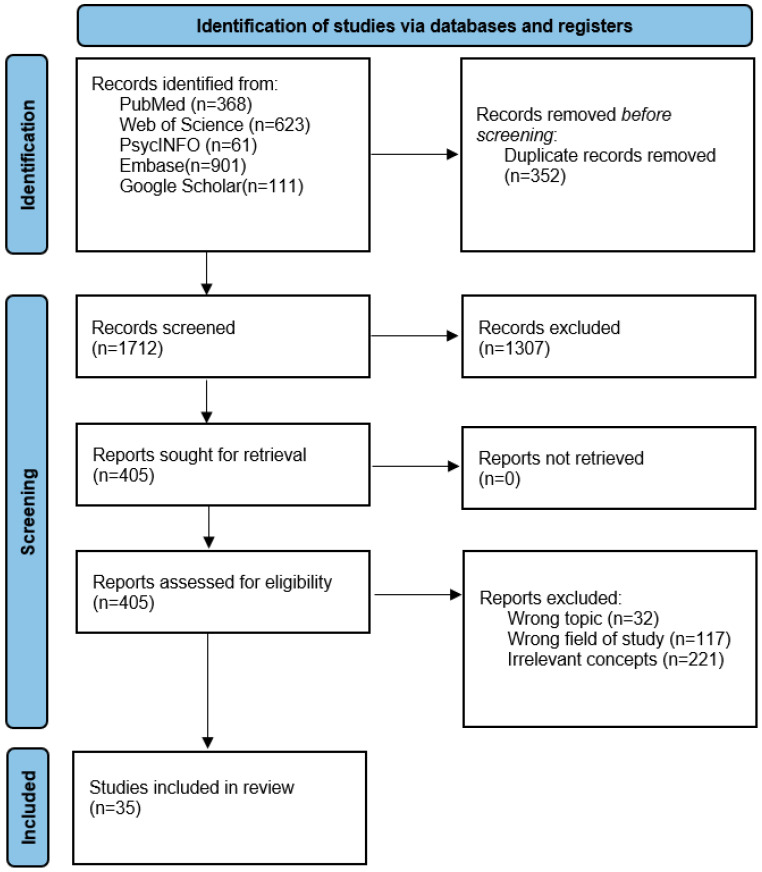
PRISMA (Preferred Reporting Items for Systematic Reviews and Meta-Analyses) flowchart for the inclusion of studies.

To conduct a thorough review of cyberpsychopathy, the PRISMA for Scoping Reviews (PRISMA-Sr) technique was used. Additionally, the technique adhered to the integrative review methodology created by de Souza and colleagues (De Souza et al., 2010). The formulation of the research topic, literature search, data collection, critical analysis of the included articles, discussion of the results, and presentation of the integrative perspective of the included articles are the six steps in this methodology (De Souza et al., 2010). A librarian with expertise in mental health assisted in the search strategy. Articles published from the databases’ inception until December 2024 were retrieved by searching Medline, Embase, Web of Science, PsycNet (PsycINFO), and the Google Scholar search engine. Search terms were used to encompass relevant concepts such as cyber, internet, digital, psychopathy, online, and narcissism. All the authors conducted the literature search. Titles/abstracts and full texts were screened by at least two reviewers working independently and blinded to each other’s decisions. A third reviewer acted as an arbitrator when disagreements arose. Conflicts comprised 9.1% of title/abstract records and 7.4% at the full-text stage; all were resolved by consensus after discussion. Inter-rater reliability was *κ* = 0.82 (titles/abstracts) and *κ* = 0.79 (full texts), indicating substantial agreement. The updated PRISMA flow diagram (Figure 1) reflects these numbers. Supplementary Materials has comprehensive search strategies and additionally includes the PRISMA-Sr checklist. The study was not previously registered.

### 2.2. Study Eligibility

If the examined papers satisfied the following inclusion requirements, they were added to the analysis: (1) the main topic of the study was on violent behavior in cyberspace (internet or digital application); (2) the study was conducted in the field of psychiatry, psychology, or social sciences; (3) one or more concepts about violence in the cyberspace is associated with another concept; and (4) French or English was the main language used in the identified manuscript. The analysis did not include case studies, protocols, pre-experimental research, or unpublished works.

### 2.3. Data Extraction

Two reviewers independently and blindly extracted study characteristics into a pilot-tested Excel workbook containing locked drop-down menus and conditional formatting, capturing five a priori fields: authors (first author and year), sample (size, age/sex, country, and special population), concepts (psychosocial constructs or online behaviors such as “online disinhibition” or “grandiose narcissism”), associations between concepts (effect sizes or qualitative linkages), and main outcomes (key findings, with quotations where possible). After side-by-side comparisons of the spreadsheets, red-flagged discrepancies were resolved by consensus, with a third reviewer adjudicating as needed, which led to 99.2% agreement (*κ* = 0.81); the cleaned dataset appears in the Supplementary Materials.

### 2.4. Data Analysis

Considering this study attempted to define the entity known as cyberpsychopathy, the biopsychosocial model was followed to classify the different concepts identified during the analysis of the studies (Bolton, 2023). The frequency of each concept as well as the reported associations between the concepts were used to establish the major concepts and subconcepts encompassed in the definition of cyberpsychopathy.

The biopsychosocial model is a thorough framework for comprehending health and illness as a result of the complex interactions between biological, psychological, and social elements (Engel, 1977). The field of psychiatry, including forensic psychiatry, makes extensive use of it.

Genetic predispositions, neurological functions, physiological reactions, and general physical health are examples of biological influences in this model. For instance, mental and physical disorders might be influenced by hormone imbalances and genetics (Molina, 1983). Psychological elements include personality traits, emotions, coping strategies, and cognitive processes. Individual well-being is greatly influenced by mental health issues, cognitive processes, and stress reactions (Papadimitriou, 2017). Finally, the social factors encompass social structures, relationships, cultural norms, socioeconomic status, and environmental impacts (Papadimitriou, 2017). All these elements are pertinent when defining the components of psychiatric illnesses and were used to shape the conceptual framework of cyberpsychopathy.

To guide thematic synthesis, a qualitative content analysis approach grounded in the biopsychosocial model as a sensitizing framework was conducted. All extracted “Concept” and “Main Outcome” fields were imported into QDAMiner for coding. Using an open coding strategy, two reviewers independently labeled recurrent constructs across studies, such as anonymity, impulsivity, narcissism, boredom, moral disengagement, or online reinforcement. These first-order codes were compared, merged, and clustered through an axial coding process to identify higher-order categories reflecting biological (e.g., traits and impulsivity), psychological (e.g., motivations and emotional regulation), and social (e.g., environmental affordances and cultural norms) components. Thematic clusters were iteratively refined into six distinct domains: online behaviors, online environments, sociodemographic factors, personality traits, psychological factors, and motivations.

Thematic saturation was assessed through constant comparison. No new codes or categories emerged after coding 85% of the dataset (30/35 studies), and the remaining 5 studies fit entirely within the existing coding structure. Final inter-coder agreement, measured on a random 10% subsample, reached *κ* = 0.81.

### 2.5. Quality Assessment

Two well-known instruments were used to evaluate the quality of the studies that were part of this analysis: the Cochrane Risk of Bias Tool for randomized controlled trials and the Newcastle–Ottawa Scale for nonrandomized controlled studies (Higgins et al., 2011; Stang, 2010).

The Cochrane Risk of Bias Tool was used to comprehensively evaluate potential biases in randomized controlled trials. Random sequence creation, allocation concealment, blinding of participants and staff, blinding of outcome assessments, completeness of outcome data, selective reporting, and other possible sources of bias are the seven domains that are examined by this technique (Higgins et al., 2011). According to predetermined criteria, each domain is classified as having a low, high, or uncertain risk of bias. In order to assess the quality of cohort and case–control studies, the Newcastle–Ottawa Scale looks at three important areas: research group selection, group comparability, and exposure or outcome determination. Studies receive stars for fulfilling the requirements for each domain; the best quality is represented by a maximum score of nine stars (Stang, 2010).

The studies in this review were divided into three quality categories: low-quality studies were those with 1–3 stars on the Newcastle–Ottawa Scale or those that were rated as having a high risk of bias using the Cochrane tool; moderate-quality studies were those with 4–6 stars or a moderate risk of bias; and high-quality studies were those with 7–9 stars on the Newcastle–Ottawa Scale or a low risk of bias.

## 3. Results

### 3.1. Description of Studies

A total of 2064 records were retrieved through the initial search. After removing 352 duplicate entries, 1712 unique studies remained. Following title and abstract screening, 1307 articles were excluded due to a lack of relevance. Among the remaining studies assessed in full, 32 were excluded for not addressing the appropriate topic, 117 were found to fall outside the fields of psychiatry, psychology, or social sciences, and 221 did not establish a clear link between the concept of cyber-related violence and another relevant construct. After a thorough study of the articles chosen for eligibility evaluation, a total of 35 articles were kept in full. Figure 1 provides specifics of the article selection procedure, and the papers that were found, as well as their quality assessment, are listed in Supplementary Materials.

### 3.2. Main Concepts

**Figure 2:**
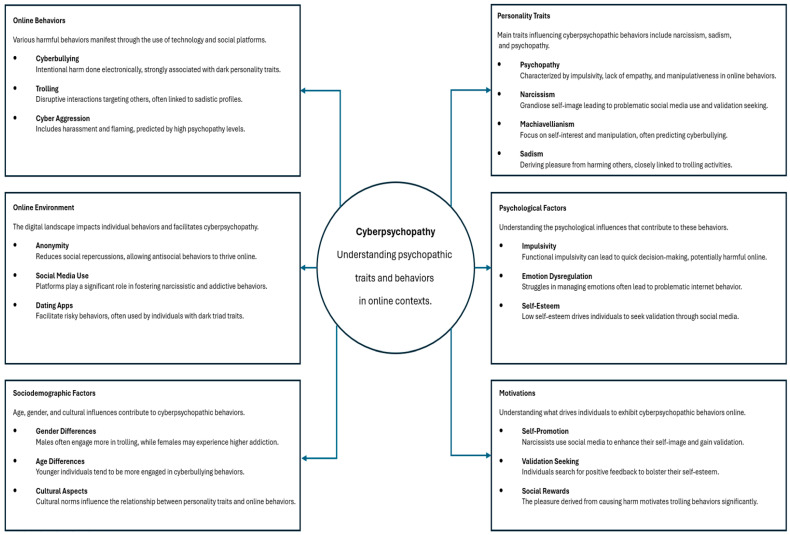
Conceptual framework for cyberpsychopathy.

From the analysis of the 35 identified studies, six central conceptual domains were identified as core constructs for defining cyberpsychopathy: online behaviors (n = 15), online environments (n = 11), sociodemographic factors (n = 7), personality traits (n = 24), psychological factors (n = 12), and motivations (n = 3). These domains were derived to capture the multifaceted nature of cyberpsychopathy as a phenomenon situated at the intersection of personality, digital affordances, social context, and internal psychological states. This framework provides a comprehensive lens through which the expression and development of psychopathic traits in online environments can be systematically examined. A conceptual framework for cyberpsychopathy is presented in Figure 2. Conceptual domains and representative variables are found in Table 1.

#### 3.2.1. Online Behaviors

A significant portion of the literature explores the link between antisocial or maladaptive online behaviors and psychological or personality traits. Several studies focus on cyberbullying and online trolling as key behavioral outcomes. Brown et al., studying 1464 social media users (mean age = 22.48), found that narcissism significantly predicted cyberbullying behaviors, alongside other dark traits (Brown et al., 2019). Similarly, Buckels et al., using a U.S. adult sample (n = 345), reported that online trolling was strongly associated with sadistic traits, arguing that sadists find intrinsic pleasure in online cruelty (Buckels et al., 2019). Smith et al. also developed one of the foundational cyberbullying assessment tools, enabling researchers to quantify online aggression consistently across studies (P. K. Smith et al., 2008). Maftei et al. explored cyberostracism and found that adolescents with higher levels of narcissism and emotional instability were more reactive to online exclusion (Maftei & Pătrăușanu, 2024).

Social media use is another central behavior. Andreassen and colleagues analyzed a massive sample (n = 23,532) and found that addictive social media use correlated strongly with narcissism (β = 0.35, *p* < 0.001) and low self-esteem (β = −0.29, *p* < 0.001) (Andreassen et al., 2017). Similarly, Brailovskaia et al., in a sample of Facebook users (n = 327), showed that both vulnerable and grandiose narcissism were significant predictors of Facebook addiction (βs ranging from 0.24 to 0.38, *p* < 0.001) (Brailovskaia et al., 2020). Kircaburun et al. demonstrated that Instagram addiction is associated with both narcissistic traits and low self-control, reinforcing the role of personality and impulsivity in shaping online behavioral addictions (Kircaburun et al., 2021). Online sexual behaviors also emerged in Ciocca et al., where sociosexuality (β = 0.41) was the strongest predictor of casual sex via Tinder (Ciocca et al., 2020).

Galica et al., who examined how problematic internet use is associated with interpersonal difficulties, interestingly suggested that individuals may engage in maladaptive online behaviors to cope with offline relational stress (Galica et al., 2017). Giumetti et al. found that workplace cyberbullying behaviors were linked to displaced aggression and low agreeableness, extending the concept of online deviance to professional environments (Giumetti et al., 2022). Moreover, Kuss et al.’s study on online gaming addiction identified patterns of excessive use linked to poor psychological well-being and escapism motives (Kuss & Griffiths, 2011).

These studies illustrate how online behaviors, ranging from social media addiction to trolling and dating app use, serve as observable outcomes of deeper psychological and personality-based mechanisms.

#### 3.2.2. Online Environment

The online environment plays an important role in shaping psychological tendencies and behavioral outcomes, particularly in individuals with vulnerable or antisocial personality traits. Across several studies, it is suggested that the structure of the digital world can lead to traits like impulsivity, narcissism, and disinhibition.

Aboujaoude et al. proposed that the internet can contribute to a broad psychological shift by amplifying maladaptive traits, particularly impulsivity and narcissism (Aboujaoude, 2017). In their review of the literature, they reported a large-scale study (n = 12,521) that compared internet users and non-users and suggested that the immediacy, anonymity, and reward structures of digital platforms reinforce narcissistic behaviors and reduce empathy. Though specific effect sizes are not provided, the study’s extensive sample size across multiple populations (e.g., gamblers and general users) lends robust support to the hypothesis that the internet is not a neutral tool but a transformative psychological space.

Kasper et al. explored how the online environment interacts with pornographic content consumption and narcissistic traits (Kasper et al., 2015). In a sample of 257 adults (mean age = 29), they found that individuals high in narcissism were significantly more likely to consume internet pornography and reported greater sexual permissiveness. The study reported that those who had watched internet pornography had higher narcissism scores than those who had never watched it (exact statistical values not provided in the grid) (Kasper et al., 2015). These findings support the idea that online sexual content may serve as a behavioral reinforcement loop for narcissistic individuals that is facilitated by the anonymity and accessibility of digital content.

Leite et al., using a sample of 773 adults (aged 19–78), analyzed the interaction between dark personality traits (particularly Machiavellianism) and problematic internet use, including behaviors like cyberstalking and doxing (Leite et al., 2023). They found that online disinhibition significantly increased the relationship between dark traits and cyberstalking behaviors, especially in female participants. Specifically, being female was positively associated with cyberstalking, and online disinhibition enhanced this effect among individuals scoring high on Machiavellianism. These findings highlight how the structure of the online environment (e.g., enabling disinhibition, secrecy, and access to personal data) facilitates the enactment of manipulative or controlling behaviors.

#### 3.2.3. Sociodemographic Factors

Sociodemographic variables, particularly gender, age, and education level, are crucial in understanding how individuals engage in various forms of online behavior and how these behaviors interact with personality traits.

Kristinsdottir et al., studying a sample of 334 adults (66.7% male), found that all three types of narcissism (agentic, communal, and vulnerable) were positively associated with social media use, with communal narcissism showing a slightly stronger correlation (Kristinsdottir et al., 2021). While effect sizes were not explicitly stated, this study suggests that the male gender was overrepresented in higher narcissism scores, particularly for agentic narcissism, indicating gender-based expression of narcissistic traits online.

March et al. conducted a study on 400 participants (67.5% women) to examine the role of gender and personality traits in predicting trolling (March & Steele, 2020). The authors found a significant positive relationship between the male gender, high sadism and psychopathy, and trolling behavior. Also, in the context of online moral behavior, Muir et al. analyzed data from 411 participants, including diverse gender identities (Muir et al., 2023). They found that moral disengagement, emotional reactivity, and moral grandstanding predicted online shaming behaviors. Together, these variables explained 23% of the variance in online shaming behavior (R^2^ = 0.23), suggesting that sociocognitive traits interact with digital behaviors in a gender-diverse sample. Lui et al. found that younger males were more likely to engage in anonymous antisocial online acts, suggesting gender and age as moderators of digital disinhibition (Lui et al., 2019).

Saulnier et al. focused on adolescents and young adults (n = 392), revealing that moral identity and moral disengagement vary with online engagement, especially in family and peer contexts (Saulnier & Krettenauer, 2023). Though specific age-related trends were not detailed, the age distribution (mean age = 19.8 years) supports the idea that younger users are particularly vulnerable to shifts in moral self-concepts when online.

Volkmer et al., in a large sample of 1026 German university students (mean age = 23.8), found that not all Dark Tetrad traits were equally associated with trolling behavior, and that gender moderated some of these associations, with men scoring higher on psychopathy and sadism (Volkmer et al., 2023). This emphasizes that sociodemographic profiles interact with personality in shaping digital deviance.

Overall, these studies reveal that gender (often male), young age, and, in some cases, lower education levels are consistently linked to higher engagement in antisocial or morally questionable online behavior. These factors often moderate the expression of personality traits and psychological vulnerabilities in digital environments.

#### 3.2.4. Personality Traits

The vast majority of the identified studies explore the role of personality traits, particularly the Dark Triad/Tetrad (narcissism, Machiavellianism, psychopathy, and sadism), in predicting maladaptive online behaviors and tendencies. These traits often serve as core explanatory variables for phenomena such as trolling, cyberbullying, problematic social media use, and online aggression.

Narcissism is the most frequently studied trait. Meta-analytic evidence from a study by Gnambs et al., which reviewed 57 studies, confirms a small-to-moderate positive association between narcissism and social networking behavior, particularly with agentic and grandiose narcissism (Gnambs & Appel, 2018). Andreassen et al., in a large-scale Norwegian sample (n = 23,532), reported a strong correlation between narcissism and addictive social media use (β = 0.35, *p* < 0.001) (Andreassen et al., 2017). Brailovskaia et al. similarly found that vulnerable and grandiose narcissism significantly predicted Facebook addiction (βs ranging from 0.24 to 0.38) (Brailovskaia et al., 2020). These results suggest that individuals with narcissistic traits use social media to regulate self-esteem and seek admiration.

Other traits of the Dark Tetrad also play important roles. Brown and colleagues showed that all four traits (narcissism, Machiavellianism, psychopathy, and sadism) predicted cyberbullying among 1464 social media users (Brown et al., 2019). Buckels et al. found that sadism was the strongest predictor of online trolling, emphasizing the unique role of this trait in promoting cruel online behaviors (Buckels et al., 2019). Lyon’s study highlighted the interplay between social dominance orientation and digital aggression, providing a sociopolitical angle on personality-driven behaviors online (Lyons et al., 2022). Volkmer and March further confirmed these associations, noting that male gender and higher sadism/psychopathy scores are significantly related to trolling tendencies (March & Steele, 2020; Volkmer et al., 2023). Heyman et al. found that higher levels of callous–unemotional traits were predictive of online manipulation and catfishing behaviors in emerging adults (Heyman et al., 2022).

Ciocca and team extended the literature to dating app usage, demonstrating that sociosexuality (β = 0.41), linked with narcissistic traits, was the main predictor of casual sex via Tinder (Ciocca et al., 2020). Also, Leite et al. found that online disinhibition mediated the relationship between Machiavellianism and cyberstalking, with women particularly affected (Leite et al., 2023).

Traits related to self-regulation and emotional instability are also highlighted in a study by Bui et al., which showed that borderline personality disorder traits and insecure attachment styles predicted higher susceptibility to online victimization and aggression (Bui & Pasalich, 2021). Rogier et al. revealed that pathological narcissism, especially the grandiose type of narcissism, was significantly associated with social media addiction (Rogier et al., 2022). Also, Schokkenbroek et al., working with over 1100 Belgian adults, highlighted the moderating role of self-control in the link between dark traits and online behavior (Schokkenbroek et al., 2024).

Finally, studies by Liu et al., Lee et al., and Park et al. linked narcissism with selfie posting, privacy disregard, and functional impulsivity, revealing how narcissistic and impulsive traits manifest in subtle online self-presentation and risk-taking behaviors (Liu et al., 2013; Lee & Sung, 2016; Park et al., 2024).

These findings strongly support the notion that personality traits (especially the Dark Triad/Tetrad and emotional dysregulation patterns) are constructs in understanding problematic and antisocial online behavior. They not only predict direct behaviors like trolling or addiction, but also explain more nuanced forms of online expression, from narcissistic status updates to manipulative interactions in dating apps.

#### 3.2.5. Psychological Factors

Psychological factors such as self-esteem, emotional dysregulation, and oversensitivity are frequently implicated in problematic online behaviors, either as precursors to or consequences of these behaviors. While often interwoven with personality traits, these dimensions reflect broader affective and cognitive vulnerabilities that manifest in digital contexts.

Self-esteem is one of the most consistently studied variables. Andreassen et al. found that low self-esteem was significantly associated with addictive social media use (β = −0.29, *p* < 0.001), suggesting that individuals may use online platforms to regulate fragile self-concepts (Andreassen et al., 2017). This is reinforced by Brailovskaia et al., who demonstrated that both vulnerable and grandiose narcissism predicted Facebook addiction again, pointing to self-esteem regulation as a core mechanism (Brailovskaia et al., 2020). Similarly, Hussein et al. reported that self-esteem was negatively associated with problematic social networking use, further emphasizing its protective role (Hussain et al., 2021).

Emotional vulnerability also appears to be a relevant factor. Buckels et al. and Marrington et al. both examined emotional dysregulation and its association with trolling (Buckels et al., 2019; Marrington et al., 2023). In adolescents, Marrington et al. found that low empathy and high emotional reactivity were significantly related to engagement in trolling. They highlighted that trolling is closely tied to sadism, a trait often associated with poor emotion regulation (Marrington et al., 2023). Fegan et al., working with a smaller sample (n = 115), explored the role of oversensitivity and attachment style in social media overuse and proposed that oversensitivity (to criticism or exclusion) contributes to compulsive digital behavior (Fegan & Bland, 2021).

March et al. also integrate psychological traits into a broader model, noting that low self-esteem and high sadism/psychopathy were significant predictors of online trolling (March & Steele, 2020). Their findings support a model in which vulnerable self-concept and emotional coldness may interact, leading to digital aggression.

In the adolescent context, Resett et al. reported that emotional dysregulation was significantly associated with both traditional and cyberbullying among high school students (n = 898), with a notable portion of the sample exclusively engaging in cyberbullying (7.6%) (Resett & Gamez-Guadix, 2017). Soares et al. showed that perpetrators of antisocial online behaviors (e.g., harassment and bullying) often display a triad of risk factors: affective instability, low self-esteem, and lack of social connectedness (Soares et al., 2023).

#### 3.2.6. Motivations

Although relatively underrepresented in the dataset, studies examining motivations behind online behaviors reveal important insights into the underlying drivers of cyberaggression, harassment, and other antisocial digital conduct. These motivations often operate alongside personality traits and psychological factors, shaping how and why individuals choose to behave aggressively online.

Nocera et al., studying 317 college students, examined intentional harm via electronic means, or cyberaggression, and how it relates to both personality and motivational variables. They found that each of the Dark Triad traits (narcissism, Machiavellianism, and psychopathy) positively correlated with cyberaggressive behaviors, but crucially, the effect was mediated by motivational factors, such as the desire to exert control or retaliate (Nocera & Dahlen, 2020). Although precise coefficients are not reported in the grid, the study emphasized that individuals with higher dark traits were more likely to report “getting back at someone” or “making someone feel bad” as primary reasons for their behavior. This points to motivations that are not purely impulsive or reactive but goal-directed and instrumental, fitting with the manipulative and strategic nature of dark personalities.

### 3.3. Quality Appraisal

Of the 35 eligible studies, 12 were rated as high quality, 20 as moderate quality, and 3 as low/unclear quality, and the appraisals of individual studies are reported in the Supplementary Materials. Online environment mechanisms (e.g., anonymity, algorithmic reinforcement, and disinhibition) were examined in 8 of the 12 high-quality papers and two controlled experiments, yielding consistent evidence that platform affordances amplify psychopathic or Dark Tetrad traits online. By contrast, sociodemographic moderators (age, gender, education, and culture) were explored mainly in moderate-quality cross-sectional surveys and narrative reviews (15/20 moderate; 2/3 low), producing mixed and sometimes contradictory associations; only 2 high-quality studies provided culturally nuanced analyses. Consequently, the overall confidence in the association of cyberpsychopathy concepts is graded as moderate: findings related to online environments are supported by a largely high-quality evidence base, whereas conclusions about sociodemographic influences remain provisional and hypothesis-generating. Readers should therefore treat the online environment domain as comparatively well-established while viewing sociodemographic patterns as preliminary signals that warrant confirmation through future longitudinal and experimental research.

### 3.4. Definition of Cyberpsychopathy

Combining the results, cyberpsychopathy is conceptualized as a digitally mediated syndrome characterized by manipulative behaviors (e.g., trolling), emotional detachment, and reward-seeking, which are amplified by platform design features like algorithmic reinforcement. It includes a variety of online actions that demonstrate a lack of empathy, manipulativeness, impulsivity, and a disrespect for social standards, including cyberbullying, trolling, deception, and cyberaggression. These behaviors are specifically shaped and enabled by the online environment, where characteristics such as anonymity, continuous accessibility, and algorithm-driven social reinforcement increase disinhibited behavior and decrease accountability.

Sociodemographic variables such as age, gender, and cultural background affect the manifestation of cyberpsychopathy by varying the frequency, type, and severity of these behaviors. Fundamentally, cyberpsychopathy stems from persistent psychological qualities that make people more likely to take advantage of others for their own selfish or malevolent purposes in digital settings, particularly those of the Dark Tetrad (psychopathy, narcissism, Machiavellianism, and sadism).

Psychological traits like impulsivity, mood dysregulation, and low self-esteem, in addition to dispositional factors, are important in the initiation and maintenance of cyberpsychopathic activities. Certain incentives, such as the need for approval, social dominance, emotional fulfillment, and identity improvement, further promote these actions.

Overall, cyberpsychopathy is best viewed as a functional construct that captures how psychopathic dispositions are enacted and amplified online, rather than a separate clinical diagnosis or personality disorder. This construct helps in comprehending how antisocial personality traits combine with the technological advantages of the digital world to produce a new, widespread, and socially significant type of psychological pathology.

## 4. Discussion

### 4.1. Principal Results

This integrative review sought to identify the principal concepts that define the phenomenon of cyberpsychopathy and to offer an initial operational definition based on these findings. A total of 35 studies met the inclusion criteria and were analyzed in depth using a biopsychosocial framework. From this analysis, six central conceptual domains emerged: online behaviors (n = 15), online environments (n = 11), sociodemographic factors (n = 7), personality traits (n = 24), psychological factors (n = 12), and motivations (n = 3). These domains collectively capture the dispositional, contextual, and psychological underpinnings of cyberpsychopathic conduct. Most studies were of moderate-to-high quality based on established critical appraisal tools. The cyberpsychopathy construct diverges significantly from existing frameworks by integrating trait vulnerabilities, psychological states, functional motivations, and digital affordances into a unified explanatory model. This integration enhances clinical relevance by offering precise intervention targets and assessment strategies, thus going beyond mere descriptive associations.

### 4.2. Comparison with Prior Work

Previous reviews exploring online antisocial behavior have often focused on specific behaviors, such as cyberbullying or trolling, without embedding these within a broader personality framework. For instance, studies by Buckels et al. (2019) and Brown et al. (2019) clearly demonstrated that sadism and psychopathy are core predictors of trolling and cyberbullying, respectively. However, these works were not framed within a unified conceptual model like cyberpsychopathy. Meta-analyses by Kowalski et al. and more recently by Basharpoor et al. emphasized emotional empathy and the Dark Triad as correlates of cyberbullying, but they did not systematically integrate motivational, environmental, or demographic moderators of these behaviors (Kowalski et al., 2014; Basharpoor et al., 2024). In contrast, our review expands this field by showing how multiple domains (including online affordances, self-regulation, and user motivations) converge to express what we propose as cyberpsychopathy, thus offering a novel biopsychosocial conceptualization.

Furthermore, while existing reviews like that by Gioia et al. have highlighted emotional dysregulation as a contributing factor in problematic internet use, they did not link such psychological vulnerabilities directly to antisocial online behavior (Gioia et al., 2021). Our findings show that traits such as impulsivity and low self-esteem not only increase the risk of problematic use but are also closely tied to manipulative and aggressive behaviors online, especially when combined with narcissistic and Machiavellian tendencies. This is consistent with findings from Schade et al., who used structural equation modeling to link dark personality traits to cyberbullying through empathy and emotional intelligence deficits (Schade et al., 2021). However, our review extends this line of inquiry by showing that these relationships are mediated by the architecture of online environments, such as anonymity and disinhibition, reinforcing the need to consider contextual affordances.

In contrast to earlier studies that often used a narrow demographic (e.g., adolescents or college students), our review highlights the importance of sociodemographic variability in cyberpsychopathy. For instance, Kristinsdottir et al. (2021) and Volkmer et al. (2023) identified gender-based differences in narcissism and trolling, findings echoed in broader research, such as that by Pineda et al., who demonstrated how the Dark Tetrad traits are differentially expressed in intimate partner cyberviolence across genders (Pineda et al., 2022). The intersectionality of age, gender, and personality traits in digital behavior has not been systematically addressed in previous studies, making our inclusion of sociodemographic dimensions a critical advancement. This richer demographic contextualization is essential for developing personalized preventive strategies and targeted interventions.

It is also important to note that the expression and societal interpretation of cyberpsychopathic behaviors are not uniform across contexts. Cultural norms determine both behavioral thresholds (e.g., what counts as “trolling” versus accepted banter) and victim-perceived harm; collectivist cultures may condemn overt aggression yet tolerate covert manipulation, whereas individualist settings show the reverse (Cook et al., 2021). Legal frameworks likewise modulate prevalence: jurisdictions with stringent cyberharassment statutes and effective enforcement report lower self-disclosed offending but higher arrest rates, suggesting that visibility and deterrence co-evolve (Adamson et al., 2023). Socioeconomic factors and digital divides further stratify risk. Limited bandwidth and shared-device environments can constrain anonymity but incentivize reputational manipulation through pseudonymous accounts, while wealthier users favor virtual private networks and niche platforms to evade detection (Khan et al., 2024). Age intersects with both access and socialization; younger cohorts, immersed in gamified ecosystems, may normalize exploitative play styles that older adults deem as antisocial (raising interpretive ambiguity when applying psychopathy)-derived constructs (Gan et al., 2024).

Also, labeling individuals as “cyberpsychopathic” carries non-trivial ethical risks. The term may reify transient, context-bound behaviors into a stable trait, fostering self-fulfilling stigma and overlooking developmental plasticity. Overpathologizing could disproportionately target marginalized groups (e.g., adolescents experimenting with identity and users from subcultures that value shock humor), thereby reinforcing digital exclusion. We therefore advocate for a cautious, behavior-first approach: clinicians, researchers, and legal actors should describe specific harmful acts, contextual affordances, and victim impact before invoking a psychopathy lens, and should couple assessment with clear avenues for remediation rather than punitive labeling.

Lastly, while previous theoretical contributions, such as Suler’s model of the online disinhibition effect, offered foundational insights into the psychology of digital behavior, these models lacked empirical validation within clinical or forensic frameworks (Suler, 2004). This integrative review not only revisits these early theories but also substantiates them with contemporary empirical studies that incorporate psychological and motivational variables. The inclusion of motivation, as seen in studies by Soares et al. (2023) and Nocera and Dahlen (2020), aligns with recent calls from authors like Basharpoor et al. to move beyond trait-based explanations and incorporate functional motives such as dominance and pleasure-seeking into predictive models of cyberaggression (Kowalski et al., 2014). Similarly, Suler’s (2004) online disinhibition hypothesis provides a valuable foundation for understanding how digital spaces lower behavioral thresholds, but it lacks the granularity to explain *who* disinhibits, *how*, and *under what psychological or motivational conditions*. Our findings suggest that disinhibition operates selectively, interacting with traits (e.g., impulsivity), states (e.g., boredom), and motives (e.g., dominance-seeking) in nonlinear ways. For example, not all narcissistic individuals troll, but those with high trait entitlement, when coupled with perceived anonymity and algorithmic reward cues, are more likely to exhibit this behavior. In this sense, our framework builds on disinhibition theory but embeds it within a trait–state–affordance interaction model. The use of the biopsychosocial model was also not merely structural but interactional. Rather than allocating behaviors to fixed domains, it explained cross-domain pathways such as how a psychological state (e.g., moral disengagement) interacts with a personality trait (e.g., sadism) and an online affordance (e.g., reward visibility) to produce digitally mediated harm. In doing so, we move from additive to emergent models of behavior, offering a more dynamic and clinically actionable account of digital antisociality than prior frameworks permit. This positions cyberpsychopathy as not merely a digital extension of traditional psychopathy but as a novel construct requiring its own diagnostic and intervention models grounded in interdisciplinary evidence.

### 4.3. Limitations

A number of limitations should be noted. First, only peer-reviewed empirical studies published in English and French were included in the review; this may have left out pertinent studies published in other languages or formats, including conference proceedings or the gray literature. Second, it was difficult to directly compare and consistently evaluate the quality of the included studies due to their diverse methodologies, which ranged from cross-sectional surveys to theoretical and neuroimaging research. Finally, the evolving nature of online platforms and user behavior means that some findings may become outdated as digital technologies and social norms continue to change. Future longitudinal and experimental studies are needed to validate and extend the proposed conceptual framework across diverse populations and digital contexts.

### 4.4. Recommendations and Implications

To translate the study’s theoretical insights into concrete action, we recommend a three-pronged strategy that aligns researchers, clinicians, policymakers, and platform designers. First, targeted digital-literacy programs should be developed for demographics shown to be most vulnerable (adolescents, older adults unfamiliar with evolving online norms, and users in low-connectivity settings) so they can recognize manipulative tactics, configure privacy tools, and seek help when confronted with cyberpsychopathic behavior. Second, social media companies can embed “friction” and prosocial nudges into interface design (for example, real-time prompts that flag potentially harmful language, reputation penalties for serial abusers, and default visibility limits on unverified accounts) to tackle the anonymity and rapid reward cycles that facilitate online exploitation. Finally, forensic and mental health services should begin piloting structured cyberpsychopathy checklists during risk assessments, coupling traditional psychopathy measures with questions about online conduct and platform affordances; this integrated approach will help clinicians identify digital risk factors early and design interventions (such as cognitive–behavioral modules on responsible online engagement) that span both offline and online domains. Together, these measures convert conceptual advances into tangible policies that reduce harm, enhance user resilience, and inform ethically grounded clinical practice.

## 5. Conclusions

In order to define and explain the newly emerging concept of cyberpsychopathy, this integrative review synthesized recent empirical data. Six interconnected conceptual domains (online behaviors, online environment, sociodemographic factors, personality traits, psychological factors, and motivations) were found through the analysis of 35 studies. Each of these domains has a distinct role in the expression of psychopathic tendencies in digital spaces. Collectively, these results suggest that cyberpsychopathy is a complex phenomenon influenced by the interaction of personal characteristics with the structural and social dynamics of digital settings, rather than just being an online manifestation of traditional psychopathy. The suggested framework and definition provide a starting point for formalizing this concept. Future research is needed to expand on these findings by creating and testing standardized instruments to evaluate cyberpsychopathy, investigating its practical ramifications, and creating focused interventions to lessen harm in online settings.

## Figures and Tables

**Table 1:** Conceptual domains of cyberpsychopathy and their representative variables.

Domain	Representative Variables	Empirical Support (# of Studies)
Online behaviors	Cyberbullying, trolling, deception	15
Online environment	Anonymity, algorithmic reinforcement, digital disinhibition	11
Sociodemographics	Age, gender, cultural background	7
Personality traits	Psychopathy, narcissism, Machiavellianism, sadism	24
Psychological factors	Emotional dysregulation, low self-esteem, oversensitivity	12
Motivations	Dominance, emotional compensation, reward-seeking	3

## Supplementary Materials

The following supporting information can be downloaded at https://www.mdpi.com/article/10.3390/ejihpe15060107/s1: Table S1: The Electronic search strategy used for the integrative review; Table S2: Preferred Reporting Items for Systematic reviews and Meta-Analyses extension for Scoping Reviews (PRISMA-ScR) Checklist; Table S3: Integrative review study selection: detailed results. Tricco et al. (2018) are cited in the Supplementary Materials.

## Abbreviations

The following abbreviations are used in this manuscript:

PRISMA-Sr	Preferred Reporting Items for Systematic Reviews—Scoping Reviews
