# Supplementary material for: Cyberpsychopathy: A Multidimensional Framework for Understanding Psychopathic Traits in Digital Environments
Source: Eur J Investig Health Psychol Educ. 2025 Jun 10;15(6):107. doi: 10.3390/ejihpe15060107 (PMC12192149; doi:10.3390/ejihpe15060107)
Supplement: Supplementary file 1 [file ejihpe-15-00107-s001.zip › ejihpe-3650065-supplementary.pdf]

Table S1. The electronic search strategy used for the integrative review.

Database; Search	Search Terms
PubMed; k= 368	("cyber" OR "internet" OR "online" OR "social media" OR "digital" OR "virtual") AND ("psychopathy" OR "antisocial personality" OR "sociopathy" OR "psychopath" OR "dark triad" OR "narcissism" OR "Machiavellianism" OR "Antisocial Personality Disorder"[MeSH]) AND Humans[MeSH Terms]
Web of Science; k= 623	TS=((cyber OR internet OR online OR "social media" OR digital OR virtual) NEAR/5 (psychopathy OR psychopath* OR "dark triad" OR narcissism OR Machiavellianism OR antisocial OR sociopathy))
PsycInfo; k = 61	((cyber OR internet OR online OR "social media" OR digital OR virtual).tw.) AND ((psychopath* OR "antisocial personality" OR "dark triad" OR narcissism OR Machiavellianism OR sociopathy).tw.)
Embase; k = 901	('cyber behavior'/exp OR 'internet'/exp OR 'social media'/exp OR "cyber" OR "internet" OR "online" OR "social media" OR "digital" OR "virtual") AND ('antisocial personality disorder'/exp OR 'psychopathy'/exp OR 'sociopathy'/exp OR "psychopathy" OR "antisocial personality" OR "sociopathy" OR "psychopath" OR "dark triad" OR "narcissism" OR "Machiavellianism")
Google Scholar; k= 111	allintitle: ("cyber" OR "internet" OR "online") ("psychopathy" OR "dark triad")

Table S2. Preferred Reporting Items for Systematic reviews and Meta-Analyses extension for Scoping Reviews (PRISMA-ScR) Checklist

SECTION	ITEM	PRISMA-ScR CHECKLIST ITEM	REPORTED ON PAGE #
**TITLE**			
Title	1	Identify the report as a scoping review.	1
**ABSTRACT**			
Structured summary	2	Provide a structured summary that includes (as applicable): background, objectives, eligibility criteria, sources of evidence, charting methods, results, and conclusions that relate to the review questions and objectives.	1
**INTRODUCTION**			
Rationale	3	Describe the rationale for the review in the context of what is already known. Explain why the review questions/objectives lend themselves to a scoping review approach.	2,3
Objectives	4	Provide an explicit statement of the questions and objectives being addressed with reference to their key elements (e.g., population or participants, concepts, and context) or other relevant key elements used to conceptualize the review questions and/or objectives.	3
**METHODS**			
Protocol and registration	5	Indicate whether a review protocol exists; state if and where it can be accessed (e.g., a Web address); and if available, provide registration information, including the registration number.	N/A
Eligibility criteria	6	Specify characteristics of the sources of evidence used as eligibility criteria (e.g., years considered, language, and publication status), and provide a rationale.	4
Information sources*	7	Describe all information sources in the search (e.g., databases with dates of coverage and contact with authors to identify additional sources), as well as the date the most recent search was executed.	4
Search	8	Present the full electronic search strategy for at least 1 database, including any limits used, such that it could be repeated.	4
Selection of sources of evidence†	9	State the process for selecting sources of evidence (i.e., screening and eligibility) included in the scoping review.	4
Data charting process‡	10	Describe the methods of charting data from the included sources of evidence (e.g., calibrated forms or forms that have been tested by the team before their use, and whether data charting was done independently or in duplicate) and any processes for obtaining and confirming data from investigators.	4
Data items	11	List and define all variables for which data were sought and any assumptions and simplifications made.	4
Critical appraisal of individual sources of evidence§	12	If done, provide a rationale for conducting a critical appraisal of included sources of evidence; describe the methods used and how this information was used in any data synthesis (if appropriate).	5

SECTION	ITEM	PRISMA-ScR CHECKLIST ITEM	REPORTED ON PAGE #
Synthesis of results	13	Describe the methods of handling and summarizing the data that were charted.	4,5
**RESULTS**			
Selection of sources of evidence	14	Give numbers of sources of evidence screened, assessed for eligibility, and included in the review, with reasons for exclusions at each stage, ideally using a flow diagram.	6
Characteristics of sources of evidence	15	For each source of evidence, present characteristics for which data were charted and provide the citations.	6-13
Critical appraisal within sources of evidence	16	If done, present data on critical appraisal of included sources of evidence (see item 12).	6-13
Results of individual sources of evidence	17	For each included source of evidence, present the relevant data that were charted that relate to the review questions and objectives.	Table S3
Synthesis of results	18	Summarize and/or present the charting results as they relate to the review questions and objectives.	10
**DISCUSSION**			
Summary of evidence	19	Summarize the main results (including an overview of concepts, themes, and types of evidence available), link to the review questions and objectives, and consider the relevance to key groups.	10,11
Limitations	20	Discuss the limitations of the scoping review process.	13
Conclusions	21	Provide a general interpretation of the results with respect to the review questions and objectives, as well as potential implications and/or next steps.	13
**FUNDING**			
Funding	22	Describe sources of funding for the included sources of evidence, as well as sources of funding for the scoping review. Describe the role of the funders of the scoping review.	13

JBI = Joanna Briggs Institute; PRISMA-ScR = Preferred Reporting Items for Systematic reviews and Meta-Analyses extension for Scoping Reviews.

* Where *sources of evidence* (see second footnote) are compiled from, such as bibliographic databases, social media platforms, and websites.

† A more inclusive/heterogeneous term used to account for the different types of evidence or data sources (e.g., quantitative and/or qualitative research, expert opinion, and policy documents) that may be eligible in a scoping review as opposed to only studies. This is not to be confused with *information sources* (see first footnote).

‡ The frameworks by Arksey and O'Malley (6) and Levac and colleagues (7) and the JBI guidance (4, 5) refer to the process of data extraction in a scoping review as data charting.

§ The process of systematically examining research evidence to assess its validity, results, and relevance before using it to inform a decision. This term is used for items 12 and 19 instead of "risk of bias" (which is more applicable to systematic reviews of interventions) to include and acknowledge the various sources of evidence that may be used in a scoping review (e.g., quantitative and/or qualitative research, expert opinion, and policy document).

From: Tricco AC, Lillie E, Zarin W, O'Brien KK, Colquhoun H, Levac D, et al. PRISMA Extension for Scoping Reviews (PRISMAScR): Checklist and Explanation. Ann Intern Med. 2018;169:467–473. doi: 10.7326/M18-0850.

Table S3. Integrative review study selection: detailed results.

Authors	Sample	Concepts	Associations between concepts	Main outcomes	Quality assessment
(Aboujaoude et al., 2017) [43]	n=12 521, Internet and non-internet gamblers<br>n=314, online and offline customers (mean age of 53 y/o)<br>n=200, French university students (mean age of 20.2 y/o)<br>n=129, undergraduate Facebook users<br>n=130 298, cross-cultural sample (Western and Eastern societies)	Online environments, personality traits (narcissism, impulsivity, aggression), obsessive–compulsive disorder models, suicide, self-promotion, regression, grandiosity	• The internet has negative effects on personality, leading to a rise in impulsivity, narcissism, and aggression due to the online disinhibition effect, self-promoting norms of expression, and an instinct-driven mode of behavior.<br>•The internet exacerbates impulse control disorders such as gambling and compulsive buying due to the immediate gratification associated.<br>•The recent rise in suicide rates is linked to the internet making	The internet nurtures negative personality traits such as impulsivity, narcissism, and aggression, leading to possible negative offline consequences (e.g., suicide, cyberbullying, regression in maturity stages, grandiosity, and exacerbation of impulse control disorders).<br>A total of 16.4% of internet gamblers are classed as moderate or severe.<br>Additionally, 17.7% of online retail shoppers meet the criteria	N/A

			self-harm impulses more difficult to resist for its users.<br>•The internet and social networking serve as vehicles for self-promotion and can therefore enhance narcissistic traits.<br>•Internet users demonstrate more gratuitous aggression, ignoring the standards of ethics and civility.<br>•Exposure to gaming violence is a predictor for offline aggression, aggressive cognition and affect, decreased empathy, and prosocial behavior.<br>•Online experience leads	for compulsive buying.	

			to regression in the adult's maturity stages because of the popular use of emojis, bitmojis, contractions, and online games.<br>•Online experience leads to unrealistic expectations, limitless optimism, and deceptive empowerment, leading to a more grandiose society.		
(Andreassen et al., 2017) [36]	n=23 532, Norwegians between 16 and 88 y/o (mean age of 35.8 y/o)	Social media use, narcissism, self-esteem	•Addictive social media use is highly related to low self-esteem, whether it serves as a predictor or a consequence, as these platforms provide safe places for them to express themselves.<br>•Addictive social	Addictive use of social media is associated with being young, female, and single.<br>Narcissism is positively correlated with addictive use of social media (r = 0.06).<br>Self-esteem is negatively	Moderate

			media is positively linked to narcissistic traits, as these platforms meet the need for affiliation and reinforce an idealized self by providing instant feedback.<br>•Young people are more impacted by social media addiction due to its role in acquiring, developing, and maintaining relationships, as well as their greater familiarity with and willingness to engage with new technologies.<br>•Women are more affected by social media addiction due to their greater	correlated to addictive use of social media (r = - 0.25).	

			susceptibility to developing addictive behaviors related to activities involving social interaction.<br>•Individuals not in a relationship may be somewhat more likely to report addictive social media use, as it offers a platform for meeting potential partners and fulfills a social function while fostering feelings of belonging.		
(Brailovskaia et al., 2020) [37]	n=327 German Facebook users (mean age of 23.67 y/o)	Self-esteem, narcissism, Facebook addiction, anxiety symptoms	•Vulnerable narcissism, grandiose narcissism, Facebook addiction, anxiety symptoms	Both vulnerable (standardized regression coefficient (SRC) 1.04) and grandiose (SRC 0.21) forms of narcissism are likely to develop addictive Facebook use.	High

				Anxiety symptoms are positively related to both vulnerable (SRC 3.00) and grandiose (SRC 0.18) forms of narcissism. Anxiety symptoms may positively mediate the association between vulnerable (and grandiose) narcissism and Facebook addiction.	
(Brown et al., 2019) [32]	n=1464 social media users (mean age of 22.48 y/o)	Cyberbullying, narcissism, Machiavellianism, psychopathy, sadism, ethnicity	•Narcissism is a predictor of cyberbullying because it serves as a defense mechanism against low self-esteem.<br>•Psychopathy is a strong predictor of cyberbullying because those with this trait are	All four traits of the dark tetrad (narcissism, psychopathy, Machiavellianism, and sadism) predict cyberbullying in participants from across three ethnicities (Asian, White, and Black).	Moderate

			found to be manipulative, pathological liars, impulsive, and unaware of or uncaring about causing others harm.<br>•Machiavellianism is a predictor of cyberbullying because of the related need to promote a perfect self on social media to gain the trust of other users.<br>•Sadism is a predictor of cyberbullying because they are more likely to be stimulated by hurting others and seeing the victims suffer, as they find it gratifying.<br>•There is a significant positive correlation		

			between mean cyberbullying and mean dark tetrad (narcissism, psychopathy, Machiavellianism, and sadism) scores.<br>•There is little ethnic variation among the dark tetrad (narcissism, psychopathy, Machiavellianism, and sadism) and cyberbullying.		
(Buckels et al., 2018)[33]	n=345 adults from the United States (mean age of 34.4 y/o)<br>n=1370 psychology students	Trolling, sadism, emotion dysregulation, moral judgment, culpability	•Trolling is closely linked to a sadistic personality profile, largely due to the increased access to the internet and the anonymity it provides, which reduces social repercussions.<br>•Both trolls and	Online trolling and sadistic personalities are strongly associated (r = 0.44-0.71). Perceived pain intensity is negatively associated with both sadism (r = -0.27) and trolling (r = -0.26).	Moderate

			sadists downplay the harm caused by their aggressive actions by employing psychological rationalization to evade feelings of guilt.<br>•Trolls and sadists react more positively and obtain more pleasure from visual representations of pain.<br>•Pleasure reactions/positive affect mediate the effects of sadism and trolling on judgments of culpability for harm.	Pleasure from pain is positively associated with both sadism (r = 0.46) and trolling (r = 0.40). Pleasure derived from pain mediates the relationship between sadism and perceived pain intensity, as well as between trolling and perceived pain intensity.	

(Bui et al., 2018) [55]	n=200 Australians (147 females; 53 males) (mean age of 22.3 y/o)	Insecure attachment, borderline personality disorder, psychopathy, psychological abuse	•Attachment anxiety and avoidance are positively associated with cyber and face-to-face psychological abuse because these individuals have a lower threshold for acting aggressively.<br>•Borderline personality disorder (BPD) traits and psychopathic traits are positively associated with cyber and face-to-face psychological abuse, as individuals exhibiting these traits tend to demonstrate dysfunctional interpersonal functioning and	BPD and psychopathic traits account for the indirect effects of an insecure attachment on face-to-face and cyber psychological abuse.<br>Attachment anxiety and avoidance are positively associated with face-to-face and cyber psychological abuse.<br>BPD and psychopathic traits are positively associated with face-to-face and cyber psychological abuse.	High

			may use manipulation to gain closeness or lack remorse and focus on control and power.<br>•BPD and psychopathic traits account for the indirect effects of attachment anxiety and avoidance on psychological abuse.<br>•BPD and psychopathic traits are significantly associated (B=0.22).<br>•Face-to-face psychological abuse is associated with cyber psychological abuse (B=0.48).		

(Ciocca et al., 2019) [39]	34 articles using different samples	Tinder use, sexual health, sexual behaviors, sociosexuality, dark triad (Machiavellianism, narcissism, psychopathy)	•Educational level is more important to women in their evaluation of male Tinder profiles.<br>•Tinder selfies are gender-dependent, with men posing to look taller and women to look curvier/more attractive.<br>•Sociosexuality (willingness to engage in sexual activity outside of a committed relationship) is the main motivator for online dating, as it offers a platform for unrestricted relationships and casual sex.<br>•Tinder users have lower sexual disgust sensitivity and	Sociosexuality is the main predictor of casual sex among Tinder users.<br>Men use Tinder mainly for casual sex.<br>Tinder is related to a lower risks of STDs.<br>Dark triad personality traits (Machiavellianism, narcissism, and psychopathy) are more frequently found in male Tinder users.	N/A

			higher sexual risqué behaviors compared to non-Tinder users.<br>• Sociosexuality is the main reason for sexual behaviors among Tinder users.<br>•There is no difference in mating success between online and offline engagement.<br>•Homosexual individuals use Tinder for relational and romantic purposes rather than for casual sex.<br>•Smartphone battery levels influence sexual decision-making and the seeking of sex.<br>•Heterosexual women consider bisexual men less attractive		

			compared to heterosexual men.<br>•Sexism is positively associated with the use of meeting apps for sexual purposes among heterosexual men.<br>•Men use meeting apps for more sexual purposes than women.<br>•The safety, misrepresentation of identities, and privacy are important concerns to American Tinder users.<br>•Beliefs associated with Tinder use vary from casual dates to sex only to marital infidelity.<br>•Women (on and off Tinder) are		

			more highly exposed to nonconsensual sex.<br>•More than 25% of encounters after a Tinder match evolve into committed relationships.<br>•Tinder is associated with a lower risk of STDs compared to other meeting apps.<br>•Use of drugs greatly increases sexual risk behaviors.<br>• The importance of time before meeting and impulsivity both influence the likelihood of sexual risk behaviors.<br>•Sexual minority men are more exposed to discrimination and violence than		

			the majority of men.<br>•Anxious attachment traits are positively associated with the use of meeting apps.<br>•Dark triad personality traits are more frequent in Tinder users than non-users.<br>•Single Tinder users are more extraverted and open to new experiences than Tinder users in a relationship.<br>•Tinder users are younger than other dating app users.<br>•Self-esteem enhancement is a predictor of problematic Tinder use.<br>•Men tend to use Tinder for sexual motives, whereas		

			women use Tinder for self-validation purposes.		
(Fegan et al., 2021) [63]	n=115 adults from Manchester Metropolitan University and the Greater Manchester area (mean age of 23.17 y/o)	Social media use, self-esteem, oversensitivity, and egocentricity	•Oversensitivity is positively associated with a higher amount of time spent online and the frequency of posts compared to egocentricity,	The authors suggest that oversensitivity is positively associated with all six aspects of social media use with greater scores compared	Moderate

			because the anxiety and preoccupation with what others think lead to the avoidance of social contacts offline, favoring social media usage.<br>•Oversensitivity is positively linked to concerns about likes and comments on social media and jealousy regarding others' posts, rather than being tied to egocentricity, due to the need for positive feedback and insecurities that drive comparisons.<br>•Both egocentricity and oversensitivity are significantly related to how	to egocentricity: time spent online (B = 0.30), frequency of posts (B = 0.24), concerns about likes/comments on social media (B = 0.46), sensitivity to negative remarks on social media (B = 0.62), jealousy related to others' posts (B = 0.50), difference in portrayal on social media compared to real life (B = 0.35).	

			different people portray themselves on social media, because vulnerable individuals lack self-esteem and tend to enhance and curate their image online in order to receive attention and approval.<br>•Egocentricity is related to less concern over negative remarks on social media compared to oversensitivity.		
(Galica et al., 2017) [40]	n=567 U.S. emerging adults (mean age of 20.0 y/o)	Conduct problems, social media use, antisocial personality disorder	•More childhood conduct problem symptoms are associated with greater daily social media use during emerging adulthood compared to antisocial personality disorder, adult-	Men report more social media use (7.41 hours) than women (5.86 hours). Symptoms of childhood conduct disorder are linked to higher social media use (B = 0.98).	Moderat e

			onset antisocial behavior syndrome (ABS), and individuals without ABS. This trend may be attributed to the anonymity that social media offers, which attracts youth with problematic behaviors.<br>•More daily social media use is associated with current antisocial personality disorder symptoms.		
(Giumetti et al., 2022) [41]	n=317 seniors from two universities in the eastern United States	Cyberbullying, cyber/traditional victimization, cyber/traditional perpetration	•Traditional victimization (TV) is strongly associated with cyberbullying victimization (CV).<br>•Traditional perpetration (TP) is strongly associated with cyberbullying perpetration (CP)	Traditional victimization is positively associated with cyberbullying victimization (B = 0.715) and predicts it in the future (B = 0.156). Traditional perpetration is positively	High

			•Machiavellianism is a predictor of CP because people who express this trait are manipulative and lack empathy.<br>•Neither affective nor cognitive empathy predicts CV or CP.<br>•CV is related to anxiety and depression because those who experience stressful negative interactions with others produce negative emotional reactions and delinquent coping responses.<br>•CV is positively associated with helping behavior, probably because victims are motivated to	associated with cyberbullying perpetration (B = 0.615) and predicts it in the future (B = 0.262). Machiavellianism is positively associated with both cyberbullying victimization (B = 0.084) and perpetration (B = 0.178) and predicts them in the future. Cyberbullying victimization leads to several negative outcomes, such as depression (B = 0.378) and anxiety (B = 0.375), but on the other hand, it leads to helping behavior (B = 0.248). Cyberbullying perpetration is	

			improve their social standing, seek to restore their connection with the community, and look forward to feeling included after a bullying encounter.<br>•CV is associated with cyberbullying perpetration, and victims are more likely to become future perpetrators.<br>•CV and CP are not related to alcohol consumption or GPAs.	strongly associated with deviant behavior (B = 0.657).	
(Gnambs et al., 2018) [52]	The meta-analysis is based on 57 studies that were published between 2008 and 2015.<br>The meta-analysis involved 25,631 participants (range of the individual samples' Ns: 31 to 2,927) from 16 countries. About 50% of all samples originated from the United States, 21% from Europe, and 15% from Asia. Approximately 60% of the participants were female, and the mean age of the samples ranged from 14 to 35 years (M: 522.82; SD: 54.72).	Narcissism, social networking behavior, culture	•There is an association between narcissism and social networking behavior (SNS). It does not vary with the platform (e.g., Facebook	The meta-analysis identified a small to moderate effect of q 5 .17 (s 5 .11), 95% CI [.13, .21], for grandiose narcissism that replicated across	Moderat e

			vs. Twitter), with the average age or gender composition of the sample, or with the year the study was conducted. It is, however, restricted to the grandiose form of narcissism. Moreover, it fluctuates with the power distance in a culture and the specific SNS behavior under study.	different social networking platforms, respondent characteristics, and time. Moderator analyses revealed pronounced cultural differences, with stronger associations in power-distant cultures. Moreover, social networking behaviors geared toward self-presentation and the number of SNS friends exhibited stronger effects than usage durations.	
(Heyman et al., 2022) [54]	n=863 undergraduate students participated in this study	Social media, positivity of impressions, narcissism, extraversion	•Greater social media use appears to be associated with liking others more, as most of	Overall, despite widespread concerns about how social media impacts in-person social	High

			the types of social media use (i.e., Instagram and Snapchat use, passive use, social network size) were positively associated with liking others.<br>•Facebook and active use, however, were not significantly associated with liking others.<br>•Overall, social media use appears to be associated with being liked more by others in a new-acquaintance context.<br>• The majority of these associations could not be explained by extraversion or narcissism, with the exception of	interactions, we found that multiple forms of social media, including Instagram, Snapchat, and passive use, were associated with more positive initial face-to-face interactions, as indicated by liking others and being liked by others more. However, other types of use, including Facebook and active use, were not significantly associated with liking in in-person interactions. Importantly, most of the associations emerged above and beyond the effects of trait-level	

			passive use, whereby extraversion appears to contribute to the relationship between passive use and being liked.	extraversion and narcissistic admiration and rivalry.	
(Hussein et al., 2021) [61]	n=555 participants (264 females; 291 males) aged between 18 and 80 years (M=33.32 years; SD=10.88); Of these, 35 participants were students, 389 were employed, 76 were self-employed, 42 were unemployed, and 13 were retired.	Problematic social networking site use (PSNSU), dark triad personality traits (Machiavellianism, narcissism, psychopathy), emotion dysregulation	• The effect of individual personal characteristics on a specific behavior is reinforced and/or mediated by affective and cognitive components, but also by difficulties in regulating emotions and in dealing with conflicts, which is illustrated as a dysfunctional coping style.<br>•Symptoms of problematic internet use were related to	The PSNSU overall score was significantly associated with dark triad traits and emotion dysregulation. Emotion dysregulation was also significantly correlated with dark triad traits. In addition, the subscales of emotion dysregulation, as well as the subfactors calculated by item parceling, were also significantly correlated. The	Moderate

			Machiavellianism and psychopathy.<br>•Emotion regulation strategies or emotion dysregulation play an important role in the development and maintenance of internet use disorders (such as PSNSU).	study highlighted the important role that emotion regulation plays in the association between dark triad traits and PSNSU.	
(Kasper et al., 2014) [44]	n =257 adults (18 - 61 y/o, mean 29), 63% female, 89% heterosexual	Narcissism and internet pornography use	•Given the sexual issues associated with narcissism, internet pornography may be another aspect of the internet that is appealing to a narcissist.	Significant differences were found between those who used and those who never used internet pornography for all measures of narcissism: Index of Sexual Narcissism $t(255) = 4.36$, $p\ .001$; Narcissistic Personality Inventory $t(253) = 2.40$, $p\ .05$; Pathological	Moderate

				Narcissism Inventory *t*(252) = 2.89, *p* .01. Significant differences were found in narcissism levels between those who currently use and those who do not currently use internet pornography for all measures of narcissism: Sexual Narcissism t(255) = 4.50, p .001; Narcissistic Personality Inventory *t(*253) = 3.48, p .001; Pathological Narcissism Inventory t(252) = 2.56, p .05; Index of Sexual Narcissism *r*(118) - .27, *p* .007; and Narcissistic Personality	

				Inventory *r*(117) = .25, *p* = .004 were significantly correlated with the frequency of internet pornography use.	
(Kircaburun et al., 2019) [38]	n =280 men and 492 women	Childhood emotional abuse, cyberbullying, dark personality traits	•Antisocial personality traits may be associated with childhood maltreatment, which interacts to produce various manifestations of antisocial behaviors such as cyberbullying.<br>•Men are better characterized by dark traits and are more likely to cyberbully than women<br>•Mediation by dark traits between childhood emotional abuse and	Dark personality traits are positively correlated with cyberbullying and childhood emotional abuse (CEA). Emotional abuse was directly associated with cyberbullying and indirectly associated via Machiavellianism, psychopathy, sadism, and spitefulness Among men, psychopathy was positively associated with emotional abuse but not with cyberbullying.	High

			cyberbullying is stronger in men than in women.	Narcissism was not associated with emotional abuse or cyberbullying. Machiavellianism, sadism, and spitefulness fully accounted for the relationship between CEA and cyberbullying. Among women, emotional abuse was directly and indirectly associated with cyberbullying. All five dark traits were positively associated with CEA, although narcissism and sadism were not associated with cyberbullying.	

(Kristindottir et al., 2021) [46]	n =334 adults (66.7 % male; 32.3 % female)	Social media use, types of narcissism (vulnerable, agentic, communal), motives for social media use (quality, validation)	•Communal narcissism has a stronger relationship with use and sharing on visual social media vs. text-based social media.<br>•Communal narcissism is related to seeking electronic feedback, and those with it rate their own self-presented content as above average.<br>•Motives for social media use (quality, validation) mediate the relationship between narcissism and actual sharing and use of social media.	All three types of narcissism have a positive relationship with sharing behavior, as well as the importance of feedback and rating the quality of their own posts for Reddit, Instagram, and Twitter.<br>All three types of narcissism are positively associated with the use and frequency of use of Instagram and Twitter.<br>Only vulnerable narcissism is positively associated with the use and frequency of use of Reddit.<br>Communal narcissism can be predicted using Instagram and Twitter,	High

				including behaviors such as sharing on all platforms, wanting feedback, and higher ratings of self-presented content. Communal and vulnerable narcissism has a positive relationship with believing that one’s content is of superior quality and with seeking validation by sharing. Agentic narcissism does not relate to social media motives or social media sharing.	

(Kuss et al., 2011) [42]	Literature review of 43 studies	Addiction to social network sites (SNSs)	•Personality will have an impact on motivations for SNS usage and patterns.	Females appear to use SNSs in order to communicate with members of their peer group, whereas males appear to use them for the purposes of social compensation, learning, and social identity gratification. Men tend to disclose more personal information on SNSs relative to women. More women were found to use MySpace specifically relative to men. Unlike women with neurotic traits, men with neurotic traits were found to be more frequent SNS users.	N/A

				Males were more likely to be addicted to SNS games specifically, relative to females.<br>“Silver surfers” (over the age of 60 y/o) have a smaller circle of online friends that differs in age relative to younger SNS users.<br>SNSs are used for social purposes.<br>Maintenance of connections to offline network members was emphasized rather than the establishment of new ties.<br>SNS users sustain bridging social capital through a variety of heterogeneous	

				connections to other SNS users.	
(Lee et al., 2016) [59]	n =315 Korean subjects<br>(avg age 29; range 19-39)<br>(94 male; 221 female)	Narcissism, selfie	•Narcissism is positively associated with the level of involvement in the feedback (i.e., comments and "likes") they receive on their selfies.<br>•Narcissism is positively associated with the degree to which one observes other people's selfies.<br>• The higher the degree to which one observes other people's selfies, the more	Levels of narcissism did not moderate the relationship between how much one observes others' selfies and the likelihood of providing a comment or "like" on other people's selfies. Individuals who have a higher level of narcissism were more involved in other people's comments and "likes" on their own selfies.	Moderate

			likely one is to leave comments or "likes" on others' selfies. •Levels of narcissism interact with the degree to which one observes other people's selfies to predict the likelihood of leaving comments or "likes" on others' selfies. •Narcissism is associated with a) more positive attitudes toward selfie-posting behavior, as well as b) greater intention to engage in selfie-posting behavior.	Narcissism was positively associated with the degree to which one observes other people's selfies. Narcissism was unrelated to the act of providing a comment or "like" in other people's selfies. The interaction effect was significant between the degree to which someone observes other people's selfies and the likelihood of leaving comments or "likes" on others' selfies. The interaction effect was not significant between levels of narcissism and the degree to	

				which one observes other people's selfies to predict the likelihood of leaving comments or "likes" on others' selfies. There is a positive relationship between narcissism and attitude toward selfie-posting behavior, as well as intention to post selfies in the future.	

(Leite et al., 2023) [45]	n =773 participants (60.4% female)<br>19 - 78 y/o, mean 27.39<br>57.2% in a romantic relationship<br>mean age of years of education: 13.06	Internet addiction, dark triad, Machiavellianism, psychopathy, narcissism, cyberstalking, online harassment, flaming behaviors, trolling behaviors	•There were moderation effects of online behaviors between personality traits and addiction to the internet.<br>•There were associations between the variables under study and the sociodemographic variables.	Being female is associated with cyberstalking total, cyberstalking justification, cyberstalking time, and online harassment.<br>Being male is associated with flaming and trolling.<br>Age is positively associated with cyberstalking control and negatively associated with all the other dimensions (internet addiction, Dirty Dozen Dark Triad Machiavellianism, psychopathy, narcissism, cyberbullying total, cyberbullying justification, cyberbullying	Moderate

				time, online harassment, flaming behaviors, trolling behaviors), i.e., as a person ages, they exhibit fewer problematic online behaviors. Years of education are positively and significantly correlated with the Dirty Dozen Dark Triad narcissism and with the justification of cyberbullying. Machiavellianism, psychopathy, and narcissism are significantly higher in men than in women. Machiavellianism is positively associated with all dimensions of the studied	

				variables. Psychopathy is positively associated with cyberstalking total, cyberstalking control, flaming, and trolling. Narcissism is positively associated with all the dimensions, except online harassment and flaming. Machiavellianism, psychopathy, and narcissism are associated with internet addiction through online behaviors.	
(Liu et al., 2013) [58]	n =780 Facebook users aged between 13 and 18 years old from secondary schools in Singapore	Privacy concerns, disclosure of personally identifiable information, narcissism, social	•Privacy concern decreases PII disclosure.<br>•Narcissism directly increases PII disclosure and is not mediated by	Privacy concerns directly decrease PII disclosure. Narcissism increases PII disclosure only directly. Social anxiety	Moderate

		anxiety, parental mediation (active and restricted)	privacy concern.<br>•Social anxiety decreases PII disclosure indirectly by increasing privacy concerns.<br>•Active parental mediation decreases PII disclosure directly and also indirectly by increasing privacy concerns.<br>•Restricted parental mediation decreases PII disclosure only indirectly by increasing privacy concerns.	decreases PII disclosure indirectly by increasing privacy concern. Active parental mediation decreases PII disclosure directly and indirectly by increasing privacy concerns. Restricted parental mediation decreases PII disclosure only indirectly through privacy concerns.	
(Lui et al., 2019) [49]	n =260 undergraduates from the US	Types of narcissism (communal, agentic), perception of Facebook status updates (likable, successful,	•Narcissistic statements are perceived more negatively than neutral statements.<br>•Agentic narcissistic statements are viewed more	Narcissistic statements are rated more negatively than neutral statements overall and across three domains. Agentic	High

		friend-worthy)	negatively than communal narcissistic statements. •Self-reported narcissism is positively related to favorable perceptions of narcissistic statements. • Females with higher levels of narcissism rate narcissistic statements more favorably than males with higher levels of narcissism.	narcissistic statements are rated more negatively than communal narcissistic statements. Female participants rated agentic narcissistic statements from female targets more negatively than male participants. Female participants rated communal narcissistic statements from male targets more negatively than male participants. Self-reported communal narcissism of the participant was related to a more favorable rating of communal	

				narcissistic statements.	
(Lyons et al., 2021) [53]	Men (n =47 men) and women (n =133) were recruited via social media and a university participation point scheme	Dark triad, rape cognitions, social media, Twitter, priming	•There is a relationship between psychopathy and sexually violent cognitions and behaviors.	Overall, participants rated the sexist tweets as significantly less acceptable (t (179) = 20.20, p < .001), less humorous (t (179) = 9.47, p < .001), more rude (t (179) = –10.88, p < .001), and more ignorant (t (179) = –8.57, p < .001) than the neutral tweets. Although sexist tweets were rated as less acceptable, exposure to sexist social media did not have an effect on	Moderate

				rape-supportive attitudes, victim blaming, or hostile masculinity. While all three dark triad traits correlated positively with rating the sexist tweets as acceptable and humorous, the prime condition itself (exposure to sexist or neutral tweets) did not impact the outcome variables. Psychopathy was the strongest predictor of proclivity to sexual violence.	
(Maftei et al., 2024) [35]	Students from a post-secondary school (N =394) and university students (N =165) from the eastern part of Romania	Addiction, personality, social interaction, stress and coping, self-concept	•The authors suggested the mediating roles of social media addiction and nomophobia on the link between	Individuals high in narcissism might be more prone to developing these behavioral addictions, which would	High

			narcissism and stress.	further lead to increased stress levels.	
(March et al., 2020) [47]	A total of 400 participants (67.5 percent women; 32.5 percent men) were recruited through Facebook and Reddit advertisements that directed them to a confidential, anonymous online questionnaire. The only inclusion criterion was an age of q18 years.	Trolling, gender, self-esteem, psychopathy, sadism	•The authors suggested that male gender, high trait psychopathy, and high trait sadism were significant predictors of trolling. Furthermore, there was a significant interaction between trait sadism and self-esteem; at high levels of sadism, there was a significant positive relationship between self-esteem and trolling.	The results indicated a significant positive relationship between self-esteem and trolling, but only when sadism was high.	Moderate

(Marrington et al., 2023) [62]	n=157 Australian adolescents<br>40.8% male; 58% female; 0.6% non-binary	Trolling psychopathy, sadism, self-esteem, empathy (cognitive and affective), social rewards (specifically negative social potency)	The following were found in the adolescent population:<br>•There is a positive correlation between trolling and psychopathy.<br>•There is a positive correlation between trolling and sadism. The correlation remained when controlling for psychopathy. However, trolling and sadism no longer correlate when controlling for negative potency.<br>•There is a positive correlation between trolling and negative social potency.<br>• Self-esteem is not a significant correlate or	In total, 24.2% reported having been trolled in the previous year (28.1% of boys; 19.8% of girls), while 13.4% reported having trolled others in the previous year (28.1% of boys; 3.3% of girls).<br><br>There were significant positive correlations ($p<.001$) between trolling and psychopathy, sadism, and negative social potency.<br>When controlling for psychopathy, sadism and trolling still shared a significant, positive	Moderate

			predictor of trolling behavior.<br>•Cognitive empathy was a significant, negative correlate of trolling behaviors, whereas affective empathy was not correlated with trolling.<br><br>•When accounting for shared variance, gender (male>female), high psychopathy, and high negative social potency are significant predictors of trolling.<br>•Sadism is not a significant predictor of trolling.<br>•Neither dimension of empathy	correlation, $r(154) = .21$, $p = .009$; however, when controlling for negative social potency, sadism and trolling were no longer correlated, $r(154) = .02$, $p = .762$<br>There were significant negative correlations ($p < .05$) between trolling and gender (male) and trolling and cognitive empathy.<br><br>Gender, psychopathy, sadism, self-esteem, cognitive empathy, affective empathy, and “negative social potency” (i.e.,	

			emerged as a predictor of trolling.	enjoyment of antisocial rewards) combined explained 30.7% of variance in adolescents' trolling behaviors ($p < .001$)<br>When accounting for shared variance, gender was a significant, negative predictor, with boys trolling more than girls, and psychopathy and negative social potency were significant, positive predictors. However, sadism was not a unique predictor of adolescents' trolling.	

(Muir et al., 2023) [48]	n =411 (311 women; 96 men; 4 other gender)<br>Ages of 15-78 (mean 24.76)<br>Online survey recruited participants in the university and via social media	Online shaming, moral grandstanding, moral disengagement, emotional reactivity, social vigilantism, online disinhibition, Machiavellianism, narcissism, psychopathy, empathy	•Moral grandstanding, moral disengagement, emotional reactivity, social vigilantism, online disinhibition, Machiavellianism, narcissism, and psychopathy are positive predictors of online shaming.<br>•Empathy is a negative predictor of online shaming.	All variables (taken together) accounted for 27% of the variance in online shaming intentions.<br>All variables (taken together) accounted for 16% of the variance in online shaming deservedness.<br>Moral grandstanding and moral disengagement had moderate positive correlations with online shaming intentions and online shaming deservedness.<br>Emotional reactivity had a small positive correlation with perceived deservedness only.<br>Empathy is	Moderate

				negatively correlated with both online shaming intentions (small) and deservedness (medium). Social vigilantism has a medium correlation with both online shaming intentions and deservedness. Online disinhibition has a medium correlation with intention and a small correlation with deservedness. Machiavellianism has a moderate correlation with both endpoints. Narcissism has a small correlation with both endpoints.	

				Psychopathy has a moderate to large correlation with online shaming intentions, but a small correlation with perceived deservedness.<br>In the regression model, psychopathy was the largest predictor of intentions to online shame.<br><br>Other results:<br>Online shaming has no relationship to any particular demographic variables.<br>No significant relationship was found between online shaming victimization and perpetration.	
(Nocera et al., 2020) [66]	n =317<br>College students<br>(220 women; 96 men; 1 did not identify)	Cyberaggression (intentional	•Each of the dark triad traits positively relates	All measures of dark triad traits positively	High

		harm via electronic devices), dark triad traits (narcissism, psychopathy, Machiavellia nism)	to cyberaggression. •Psychopathic traits account for the most variance when considering the dark triad traits together.	correlated with cyberaggression perpetration. Psychopathic traits: the erratic lifestyle subscale was the only factor to explain the unique variance in perpetration. This is likely related to externalizing behaviors and impulsivity. Men report engaging in more cyberaggression perpetration.	

(Park et al., 2024) [60]	n=141 university students<br>63% male<br>18–30 y/o<br>Monash University Malaysia	Functional impulsivity (def: “ability to react quickly and efficiently under pressure when there is necessarily no time for more careful and deliberative thinking”)<br>Dysfunctional impulsivity (def: “tendency to make quick short-term decisions without considering their delayed and potentially negative consequences”)<br>Dark triad traits: narcissism,	•Dark Triad traits are associated with cyberbullying.<br>•Functional and dysfunctional impulsivity are associated with cyberbullying.<br>•Dysfunctional impulsivity mediates the psychopathy–cyberbullying perpetration relationship.<br>•Impulsivity does not mediate the relationship between cyberbullying and narcissism.<br>•The authors suggest that there is no correlation between Machiavellianism and impulsivity scores.	In total, 26 participants scored for Machiavellianism, 7 participants scored for narcissism, and 42 participants reported none to minimal behaviors indicative of cyberbullying.<br><br>Cyberbullying is significantly correlated with all other scales: narcissism ($p<0.05$), psychopathy ($p<0.01$), Machiavellianism ($p<0.05$), functional impulsivity ($p<0.05$), and dysfunctional impulsivity ($p<0.05$).<br><br>Machiavellianism is not	Moderate

		Machiavellianism, psychopathy cyberbullying perpetration		correlated with any of the impulsivity measures.<br><br>Psychopathy is significantly correlated with functional impulsivity ($p<0.05$). Psychopathy is significantly correlated with dysfunctional impulsivity ($p<0.01$). Regarding the relationship between cyberbullying perpetration and psychopathy, the mediating effect of dysfunctional impulsivity is statistically significant. However, there is a non-significant mediating effect of functional	

				impulsivity.<br><br>Narcissism is significantly correlated to functional impulsivity (p>0.01) but not to dysfunctional impulsivity. Regarding the relationship between narcissism and cyberbullying, the mediating effect through functional impulsivity is not significant (p=0.077).	
(Resett et al., 2017) [64]	n=898 high school students in Argentina<br>56% female; mean age =15.2 y/o	Traditional bullying, cyberbullying, emotional problems (depression, anxiety), personality profile (self-esteem, consciousness,	•There are adolescents who are involved exclusively in traditional bullying or cyberbullying.<br>•Cyberbullies and traditional bullies present different emotional	The majority (82%) of adolescents were not involved. In total, 6% were considered traditional bullies, 8% were considered cyberbullies, and 4% were considered	Moderate

		agreeableness<br>,<br>extraversion, openness to experience, neuroticism)	problems (cyberbullies present less depression and anxiety than traditional bullies and traditional/cyber bullies)<br>•The authors suggest cyberbullies and traditional bullies present different personality profiles (higher levels of neuroticism in traditional bullies than cyberbullies and traditional/cyber bullies, as well as higher levels of agreeableness in cyberbullies than traditional bullies)<br>•Cyberbullying is not merely an extension of traditional bullying.	traditional/cyber bullies (involved in both forms of bullying).<br>More males than females belonged to cyberbully groups and traditional/cyber bully status: 11% versus 5%, 6% versus 2% $\chi 2(3) = 27.55$, $p < 0.001$.<br>Significant effects of belonging to a group were found in both emotional problems and personality (Wilks' Lambda = 0.92, $F(6) = 1.93$, $\eta 2 = 4\%$, and $p < 0.06$; Wilks' Lambda = 0.82, $F(10) = 2.87$, $\eta 2 = 10\%$, $p < 0.002$, respectively)<br>Cyberbullies	

				scored low in depression ($F(2) = 3.80$, $\eta 2 = 5\%$, $p < 0.05$) and anxiety ($F(2) = 3.41$, $\eta 2 = 5\%$, $p < 0.05$) compared to traditional bullies and traditional/cyber bullies. Regarding personality, belonging to a group introduced differences in agreeableness and neuroticism. Cyberbullies scored high in agreeableness compared to traditional bullies ($F(2) = 5.77$, $\eta 2 = 8\%$, $p < 0.005$). Cyberbullies and traditional/cyber bullies showed low neuroticism compared to traditional	

				bullies ($F(2) = 4.67$, $\eta2 = 7\%$, $p<0.05$).	
(Rogier et al., 2022) [56]	n=270 adults (mean age of 41.05 y/o; 78.5% male)	Behavioral addiction (social network and Facebook addiction), pathological narcissism (grandiose narcissism, vulnerable narcissism), emotional dysregulation (alexithymia)	•Pathological narcissism (grandiose and vulnerable) is associated with Facebook addiction.<br>•There is an association between grandiose narcissism and Facebook addiction because these individuals might misuse Facebook as a means to support their grandiose image of self, and	Pathological narcissism, and especially grandiose narcissism, appears to be an important factor associated with problematic Facebook usage. Difficulties in the capacity to identify one's own emotional states may be a risk factor for Facebook addiction.	High

			because they show a lack of emotional reciprocity in Facebook interactions. •Grandiose narcissism was significantly and positively correlated with alexithymia. •Contingent self-esteem is the dimension of vulnerable narcissism that showed the strongest association with Facebook addiction, because fragile self-esteem individuals are excessively dependent on others' feedback. •Alexithymia predicted levels of Facebook addiction beyond the role of		

			pathological narcissism, because these individuals showed emotional dysregulation (which is a mechanism underlying addictions) and more interpersonal problems.		
(Saulnier et al., 2022) [50]	n=392 adolescents and young adults (mean age of 19.54 y/o; 39.6% male; 82.90%; European-Canadian)	Moral identity (family and friend context, online context), moral disengageme nt (family and friend context, online context), antisocial online behavior (pirating,	•Engagement in online contexts is related to lower moral identity and heightened moral disengagement, and there is a high prevalence of intended and performed online antisocial behaviors.<br>•Moral identity is activated to a lesser degree within online contexts compared to in-	Moral identity and moral disengagement exhibit sociocognitive effects within an online context across the ages of early developmental importance. Engagement in online contexts is related to lower moral identity and heightened moral disengagement, and online	Moderat e

		trolling, hacking)	person interactions.<br>•Moral disengagement is influential within an online setting and in face-to-face environments.<br>•Moral disengagement hinders online moral identity.<br>•Moral disengagement is the only significant and positive predictor of online antisocial behavior.<br>•Moral identity was significantly different between online and family and friend contexts.<br>•There is a linear interaction between moral disengagement contexts and social	context holds less moral valence for adolescents and young adults. There is a high prevalence of intended and performed antisocial online behaviors. Online interactions are different compared to face-to-face interactions. There is a possibility that not all forms of psychological phenomena are completely altered by online contexts, and some personality characteristics and behavior may remain cross-contextually stable.	

			desirability.<br>•Online moral identity significantly predicted online moral disengagement, which was a significant predictor of performed online antisocial behaviors.		
(Schokkenbroek et al., 2024) [57]	n=1144 Belgian adults (mean age of 47.66 y/o; 51.3% female)	Dark triad traits (Machiavellianism, narcissism, psychopathy), domestic violence (cyberdating abuse), individual self-control	•There is a significant positive association between poor self-control ability and the dark triad traits.<br>•Poor self-control ability was positively associated with cyberdating abuse perpetration (B=.13, p=0.4, 95% CI [0.005, 0.245]).<br>•Psychopathy was positively	Self-control plays an instrumental role in explaining why individuals control and monitor their partners via digital technology. Cyberdating abuse was common, as 41.7% of respondents reported that they had engaged in some of the controlling and monitoring	Moderate

			associated with cyberdating abuse perpetration (B=.41, p=.000, 95% CI [0.305, 0.505]).<br>•Three dark triad traits were indirectly related to cyberdating abuse perpetration through poor self-control.<br>•Younger people and women were more likely to engage in cyberdating abuse perpetration.	behaviors towards their partners. Personality traits of the dark triad affect self-control ability, which in turn predicts the engagement in online controlling and monitoring behavior toward partners. People with psychopathic traits could be more inclined to perpetrate cyberdating abuse, regardless of their ability to exercise self-control.	

(Smith et al., 2008) [34]	Study 1: N =92 adolescents from London, UK<br>Study 2: N =533 adolescents from UK	Cyberbullying, traditional bullying	•Frequencies of cyberbullying (22.2% in study 1; 17.3% in study 2) were less than traditional bullying (58.1% in study 2), probably given the recentness of cyberbullying.<br>•Cyberbullying was experienced and reported more outside of school than in school, because of the restriction of mobile phones in school.<br>•Older students were more often internet aggressors than younger students.<br>•Cyberbullying victims were also traditional victims, and cyberbullies were traditional bullies.<br>•Picture/video	Cyberbullying is an important new kind of bullying with some different characteristics from traditional bullying.<br>Much of cyberbullying happens outside of school.	High

			bullying had a strong negative impact on the victim.<br>•Phone calls and text messaging were more prevalent forms of bullying.<br>Older students were more likely to have cyberbullied others.<br>Girls were more likely to be victims of bullying overall and to be cyberbullied.<br>Telling was significantly more likely for victims of traditional bullying (70.2%) than cyberbullying (58.6%).<br>Being a cyberbullying victim but not a cyberbully		

			correlated with internet use.		
(Soares et al., 2023) [65]	n=359 university students from Canada	Antisocial online behavior (harassment, bullying, trolling), online disinhibition, motivation for cyberaggression, self-esteem, empathy	•There is an association between appetitive motives (pro-active aggression) and being a perpetrator. •Appetitive motives for online antisocial behavior are more important than aversive motives. •Self-control does not play a significant role in the likelihood of young people engaging in antisocial behaviors.	Three factors are associated with the perpetration of online antisocial behavior: recreation, reward, and cognitive empathy, Young people engage in online antisocial behavior for fun and feel excitement and social approval. Perpetrators of online antisocial behavior have a lower capacity to comprehend the emotions of others	Moderate

			•The prevalence of online antisocial behavior is less about the nature of the medium (online disinhibition) and more about how social interactions have moved to the online environment.<br>•There is a negative association between cognitive empathy and being a perpetrator.<br>•There is an association between reward and recreation and being a perpetrator.	The prevalence of online antisocial behavior is less about the nature of the medium (anonymity, asynchronicity) and more about the individuals involved.	

(Volkmer et al., 2023) [51]	n=1026 university students from Germany (mean age of 26.46 y/o; 77.2% female)	Dark tetrad of personality (Machiavellianism, narcissism, psychopathy, sadism), online antisocial behavior (trolling), humor style (aggressive, self-enhancing, self-defeating, affiliating, katagelasticism), social exclusion	•There is an association between trolling and aggressive and self-defeating humor because these individuals like to create conflict for their own amusement.<br>•Each of the dark tetrad personality facets correlated positively and significantly with global trolling behavior.<br>•Higher aggressive humor and self-defeating humor were significantly associated with more global trolling.<br>•Experience of exclusion did not significantly impact participants' immediate	Not all facets of the dark tetrad are equally predictive of trolling.<br>The relationship between the dark personality dimensions and trolling behavior is more nuanced than previously assumed.<br>Sadism is a better predictor of trolling than the dark triad facets because it has a stronger association with katagelasticism, and people with sadistic tendencies feel greater aggressive pleasure, which may motivate them to behave aggressively.<br>Being socially excluded has no effect on	High

			trolling motivation. •Psychopathy and sadism affect trolling motivation (with sadism being the most important predictor).	immediate trolling motivation.	

ABS: Adult-Onset Antisocial Behavior Syndrome; BPD: Borderline Personality Disorder; CV: Cyberbullying Victimization; CP: Cyberbullying Perpetration; NPI: Narcissistic Personality Inventory; PII: Personally Identifiable Information; PSNSU: Problematic Social Networking Site Use; SNSs: Social Network Sites; SRC: Standardized Regression Coefficient; STDs: Sexually Transmitted Diseases; TP: Traditional Perpetration; TV: Traditional Victimization.
